# Global status of research on radiotherapy for rectal cancer: A bibliometric and visual analysis

**DOI:** 10.3389/fpubh.2022.962256

**Published:** 2022-08-08

**Authors:** Yafei Xiao, Mengyuan Qiu, Wanting Huang, Shaowen Hu, Cong Tan, Fangmei Nan, Xiaowei Jiang, Dapeng Wu, Mengmeng Li, Quanying Li, Changjiang Qin

**Affiliations:** ^1^Department of Gastrointestinal Surgery, Huaihe Hospital of Henan University, Kaifeng, China; ^2^Ruida Pharmaceutical Clinical Medicine Postgraduate Education Innovation Training Base of Henan University, Kaifeng, China; ^3^Department of Neurology, Peking University People's Hospital, Peking University School of Medicine, Beijing, China; ^4^Department of Gynecology and Obstetrics, The First Affiliated Hospital, Zhejiang University School of Medicine, Hangzhou, China; ^5^Cell Signal Transduction Laboratory, School of Basic Medical Sciences, Bioinformatics Center, Henan Provincial Engineering Center for Tumor Molecular Medicine, Institute of Biomedical Informatics, Henan University, Kaifeng, China; ^6^Department of Pediatric Orthopaedics, The Third Affiliated Hospital of Zhengzhou University, Zhengzhou, China; ^7^Department of Radiology, Huaihe Hospital of Henan University, Kaifeng, China

**Keywords:** rectal cancer, radiotherapy, bibliometrics, CiteSpace, WoSCC

## Abstract

Radiotherapy for rectal cancer has received increasing research attention in recent years; however, no bibliometric assessment has been conducted on the progress of research in this field. This study aimed to visualize the research evolution and emerging research hotspots in the field of rectal cancer radiotherapy using bibliometric methods. Data were collected from the Web of Science Core Collection database, including countries, institutions, authors, keywords, and co-citations of references, and the CiteSpace software was used for bibliometric analysis. A total of 5,372 publications on radiotherapy for rectal cancer, published between January 2000 and January 2022, were included. An increasing trend in the number of published articles was observed. There is an overall upward trend in the number of publications published, with the US publishing the most in this field, followed by China and the Netherlands. Italian writer Vincenzo Valentini and German writer R. Sauer ranked first in terms of published articles and co-cited authors, respectively. Literature co-citation and keyword co-occurrence analyses showed that early studies focused on topics such as preoperative radiotherapy, combined radiotherapy and chemotherapy, and total mesorectal excision. In recent years, gradually increasing attention has been paid to short-course radiotherapy, x-ray brachytherapy, and stereotactic systemic radiotherapy. Burst analysis suggested that magnetic resonance (MR)-guided neoadjuvant radiotherapy studies, mechanistic studies, and clinical trials may emerge as new research hotspots. Rectal cancer radiotherapy has been widely studied and the research hotspots have considerably changed in recent years. Future research hotspots may include MR-guided neoadjuvant radiotherapy studies, mechanistic studies, and clinical trials.

## Introduction

Colorectal cancer is the third most commonly diagnosed cancer and the second major cause of cancer deaths worldwide. In 2021, colorectal cancer was diagnosed in 1,931,590 patients and caused the death of 935,173 people worldwide (1). Rectal cancer is the second most commonly diagnosed cancer in the large intestine (28%) after proximal colon cancer (42%) (2), and one-third of colorectal cancer cases involve the rectum, which poses a serious threat to the life and health of patients (3).

Given the location of the rectum within the pelvis and its relationship to the urogenital organs, the diagnosis and treatment of rectal tumors have been widely considered distinct from those tumors of the rest in the colon (4). The treatment methods for patients with rectal cancer mainly include surgery, adjuvant therapy, and multidisciplinary comprehensive therapy (3). In patients with resectable rectal cancer, the standard treatment is surgery followed by chemoradiation and postoperative adjuvant chemotherapy (5). For patients with unresectable rectal cancer, radiotherapy is considered the standard of care. The goal of radiotherapy is to downstage the primary tumor to allow for an R0 resection and to decontaminate the at-risk pelvic region, in order to reduce the rate of local recurrence and improve survival (6). With the widespread application of radiotherapy in rectal cancer, the discussion on this topic has become increasingly intensive worldwide, and related studies on radiotherapy for rectal cancer are accumulating. These studies objectively reported the developmental trends and characteristics of research in the field of rectal cancer radiotherapy. How the studies have evolved, which studies have key roles and guiding significance, and how the research trends and hotspots have changed are issues that need to be explored and have great significance for future research on rectal cancer radiotherapy.

Bibliometrics is a tool that has been widely used to quantitatively evaluate the literature and to explore trends in a research field (7). CiteSpace is a tool for visualizing and analyzing the trends and patterns in scientific papers, helping researchers in understanding network patterns in research areas and in identifying and monitoring study hotspots (8, 9). In this study, we used CiteSpace for the first time to qualitatively analyse the literature on rectal cancer radiotherapy, combining all relevant studies on rectal cancer radiotherapy and chemotherapy for >20 years, in order to elucidate the research evolution trends and research hotspots in this field.

## Data collection and research methods

### Data source and collection

On 25 March 2021, we searched the literature in the field of radiotherapy for rectal cancer using the Web of Science Core Collection (WoSCC) database for the period from 1 January 2000 to 1 January 2022. Science Citation Index Expanded, Conference Proceedings Citation Index-Science, Conference Proceedings Citation Index-Social Science & Humanities, Book Citation Index-Science, and Book Citation Index-Social Sciences & Humanities were used as data sources, and the type of publication was limited to “article”. The main search terms were “rectal neoplasm”, “rectal tumor”, “rectal cancer” or “rectum cancer”, “colorectal cancer”, “colorectal carcinoma”, “colorectal tumor”, “radiotherapy”, and “radiation”. The detailed search strategies are described in the Supplementary material. Two authors (Y.X. and S.H.) independently searched the WoSCC database for relevant literatures, downloaded and saved the remaining literature in the “full record with cited references” format, and screened for duplicates, resulting in a sample of 5,372 articles for analysis.

### Research methods

The CiteSpace software (version 5.8. R3) was selected as the main tool for the comprehensive analysis of the literature on radiotherapy for rectal cancer. The CiteSpace software is a Java-based visualization program developed by Professor Chen Chaomei of Dexrel University in the United States. The data sources of Citespace software are mainly from Web of Science database and PubMed database (10). The imported data is constructed to a visual map through the following steps: procedure, time slicing, thresholding, modeling, pruning, merging and mapping (8). It combines the three functions of bibliometrics, data integration analysis and visualization methods (11). It is used to analyse the basic literature indicators, including countries, authors, institutions, keywords, and co-citations of references, So as to systematically and clearly show the development process of a research field, the emergence of emerging research fields and predict the future research direction (12, 13). The parameters of CiteSpace were set as follows: the time period was from January 2000 to January 2022; the time slice was 1 year; the term source selected all items, a node type at a time, and TOP50 or TOP30 or TOP10 as the standard; and other settings were set to the default values. The visual knowledge graph consisted of nodes and linear connections. Each node in the graph represents a keyword, and the size of the node represents the frequency of occurrence and citation. Notably, PFNET is short for “pathfinding network”, which means pruning network. According to Chen et al., pruning is one of the main procedures of the Citespace software, and effective pruning can reduce link crossover and improve the clarity of the resulting network visualization. CiteSpace supports two common network pruning algorithms, Pathfinder and Minimum Spanning Tree (MST). Compared to MST, Pathfinder uses a more sophisticated link elimination mechanism that removes a large number of links and keeps the most important ones (8). Some other details are shown as follows: Timespan: time interval of literature data; Slice Length: setted time slicing; Selection Criteria: Top n per slice: extract the top n data of each slice to generate the final network; Network: N: number of network nodes, E: number of links; Density: network density; Largest CC: maximum co-citation or co-occurrence frequency; Pruning: network pruning method; Modularity Q (Q value): Clustering module value, it is generally believed that Q>0.3 means that the clustering structure is significant; Weighted Mean Silhouette S (S value): the average silhouette value of the cluster, it is generally considered that S>0.5 clustering class is reasonable, and S>0.7 means that the clustering is convincing.

To predict the number of articles that will be published in 2022, Microsoft Office Excel 2016 (Microsoft, Redmond, WA, USA) was used to process the data and to construct a polynomial regression model: f(x) = p0x n + p1x n-1 + p2x n-2 + p3x n-3 + … + pn.

## Results

### Quantitative analysis of basic information

#### Annual growth trend of publications

**Figure 1:**
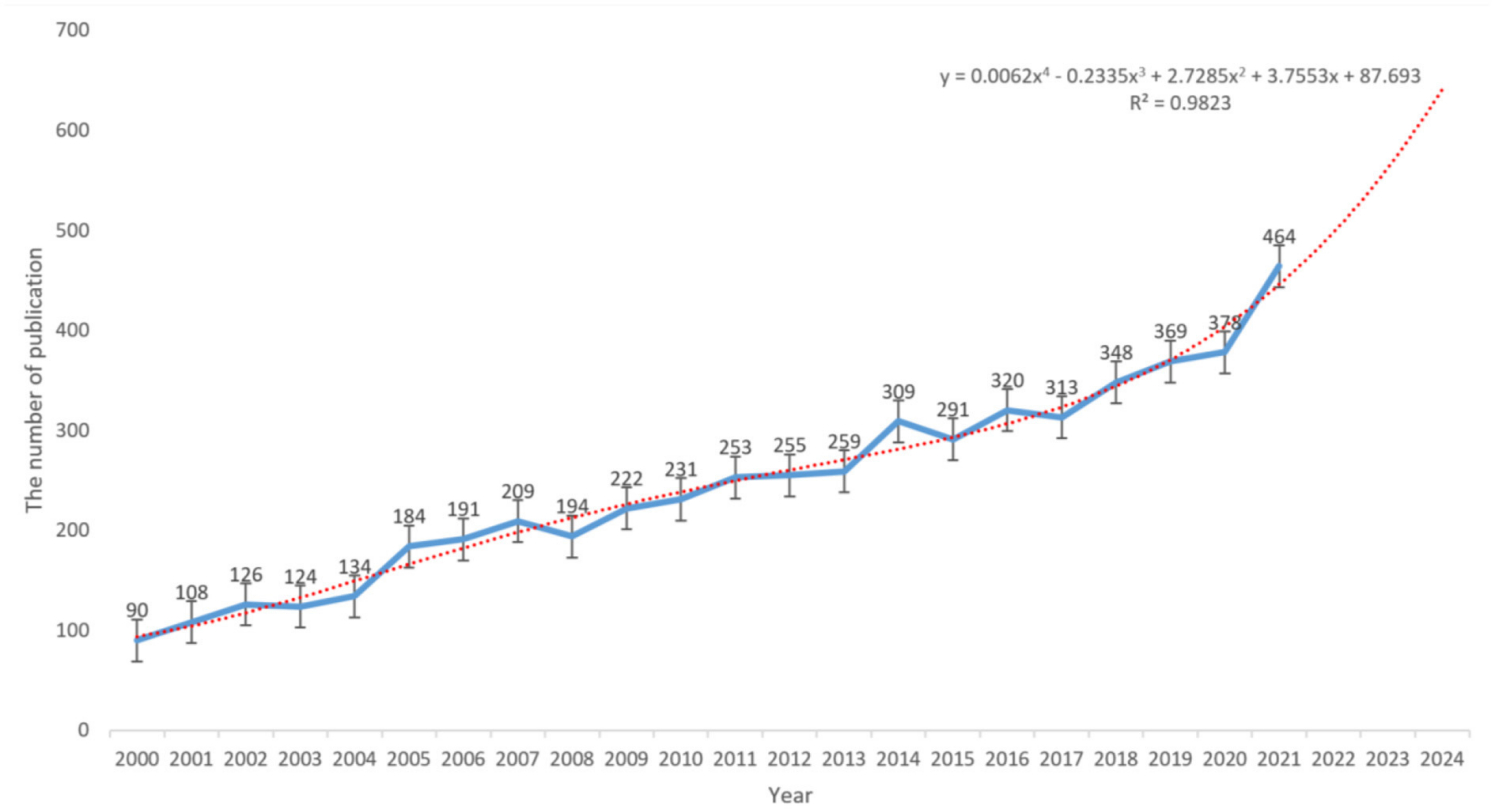
Polynomial curve fitting of publication growth in the field of radiotherapy for rectal cancer.

The search of the WoSCC database yielded a total of 5,372 articles on radiotherapy for rectal cancer published between 2000 and 2021. As shown in Figure 1, the overall trend from 2000 to 2021 was largely upward, except for small decreases in 2003 (−1.59%), 2008 (−7.18%), 2015 (−5.82%), and 2017 (−2.19%). In addition, in 2001, 2005, 2014, and 2021, the annual growth rate of literature was relatively high, which were 20.00, 37.31, 19.31, and 22.75%, respectively (Supplementary Table S1). The year with the least number of published articles was 2000 (*n* = 90, 1.68%) and that with the highest number of published articles was 2021 (*n* = 464, 8.64%), with an average of 244 articles published per year. A statistically significant correlation between the year and number of publications was observed by fitting the data (R^2^ = 0.9823). On the basis of the fitted curve, we predicted that approximately 511 articles on radiotherapy for rectal cancer will be published in 2022.

#### Country/region analysis

**Figure 2:**
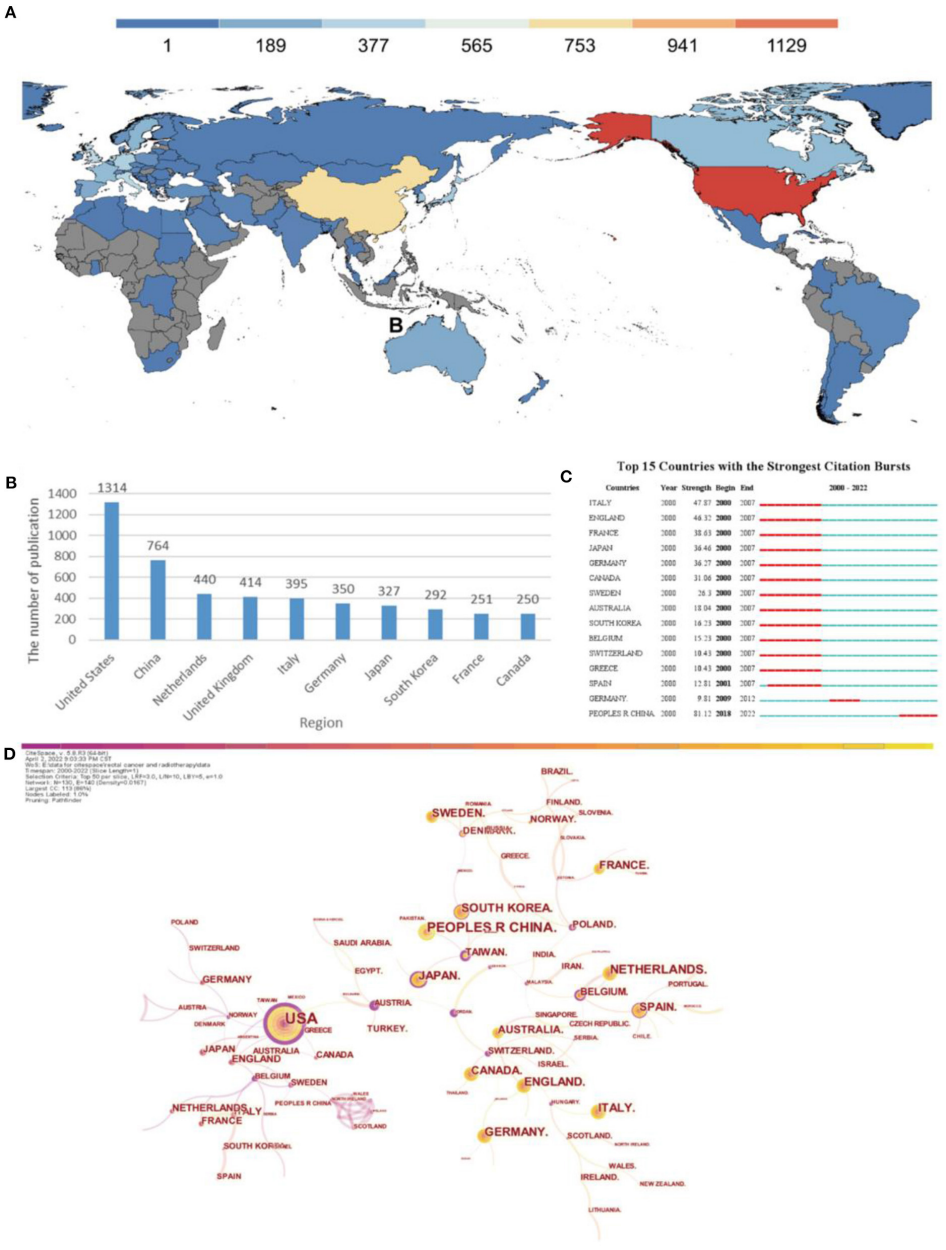
Analysis of countries engaged in research on radiotherapy for rectal cancer. **(A)** Distribution of countries in terms of publications. **(B)** Top 10 most productive countries. **(C)** Top 15 countries in publishing research related to radiation therapy for rectal cancer with burst period after 2000. **(D)** Network diagram showing country links (Timespan: 2000–2022; Slice Length: 1; Selection Criteria: Top50 per slice; Network: N = 130, E = 140; Density: 0.0167; Largest CC:113; Nodes Labeled:1.0%; Pruning: Pathfinder).

In the past 22 years, all publications on radiotherapy for rectal cancer have come from 130 countries/regions. As shown in the country distribution chart (Figure 2A) and bar chart (Figure 2B), the top 10 countries (five European, three Asian, and two North American countries) published a total of 4,797 articles, accounting for 71.47% of the total volume of publications. The top three countries/regions were the United States (*n* = 1,314, 19.58%), China (n = 764, 11.38%), and the Netherlands (*n* = 440, 6.56%) (Table 1). According to the burst analysis (Figure 2C), radiotherapy for rectal cancer was a hot topic in Europe, Japan, Korea, and Australia between 2000 and 2007. Between 2009 and 2012, research on radiotherapy for rectal cancer increased in popularity in Germany. Studies related to radiotherapy for rectal cancer have been accumulating in China since 2018, and this topic remains popular until today. In addition, many different countries/regions have shown extensive cooperation, particularly the United States, not only with the neighboring countries Mexico and Canada but also with European countries (United Kingdom, Norway, Belgium, and Greece) and Asian countries (Japan, Taiwan [China], and Australia). By contrast, China has cooperated with relatively few regions, mainly Pakistan and Sweden (Figure 2D).

**Table 1:** Ranking of institutions according to number of published articles.

**Rank**	**Count**	**Centrality**	**Year**	**Institutions**
1	137	0.15	2001	Leiden University
2	124	0.04	2000	Mem Sloan Kettering Cancer Center
3	71	0.01	2000	Catharina Hospital
4	65	0.02	2002	Karolinska Institute
5	63	0.04	2008	University Texas MD Anderson Cancer Center
6	57	0.03	2013	Fudan University
7	57	0.09	2009	Maastricht University
8	56	0.03	2015	Sun Yat Sen University
9	54	0.17	2002	Mayo Clinic
10	49	0.00	2007	Netherlands Cancer Institute

#### Institutional analysis

The top three institutions in terms of publication volume were Leiden University (*n* = 137, 6.87%), Memorial Sloan Kettering Cancer Center (*n* = 124, 6.62%), and Catharina Hospital (*n* = 71, 3.56%). Leiden University had the largest number of publications; however, the average publication year was in the early period. The institutions with more recent publications were Fudan University (n = 57, 2.86%) and Sun Yat Sen University (*n* = 56, 2.81%) in China, indicating a gradual increase in research activity related to radiotherapy for rectal cancer in China in recent years (Table 1).

**Figure 3:**
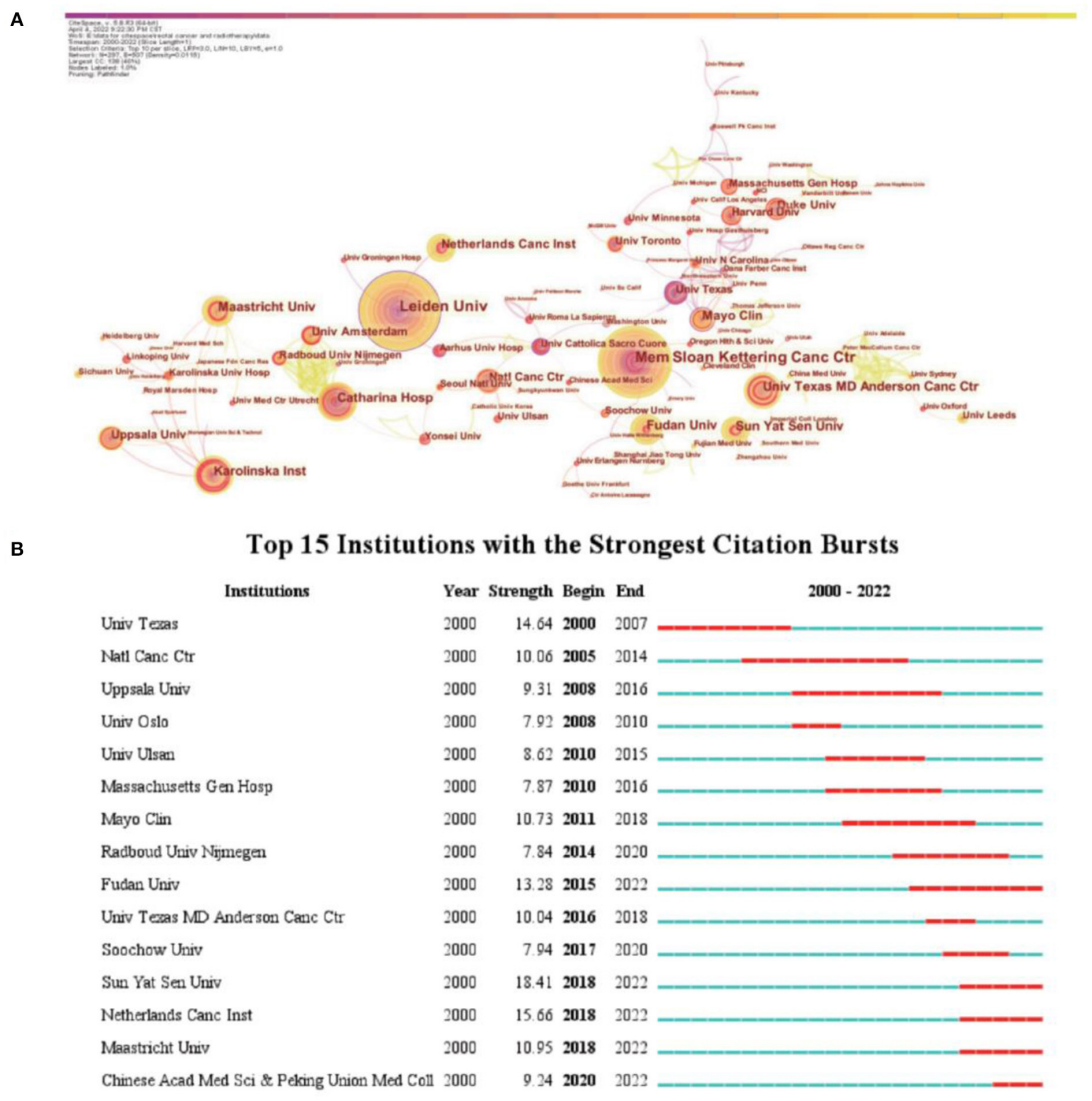
Analysis of institutions involved in research on radiotherapy for rectal cancer. **(A)** Network diagram showing institution links (Timespan: 2000–2022; Slice Length: 1; Selection Criteria: Top10 per slice; Network: *N* = 297, E = 507; Density: 0.0115; Largest CC:138; Nodes Labeled:1.0%; Pruning: Pathfinder). **(B)** Top 15 institutions in publishing research related to radiation therapy for rectal cancer with burst period after 2000.

As shown in Figure 3A, cooperation between institutions is broader than cooperation between countries. The institutions with which Leiden University cooperated were the Netherlands Cancer Institute, University Groningen Hospital, Aarhus University Hospital, and University Nijmegen. Among these, the Netherlands Cancer Institute, University Groningen Hospital, and Aarhus University Hospital more closely cooperated with Leiden University than the others. Mayo Clinic cooperated with the highest number of institutions, namely University of North Carolina, Dana Farber Cancer Institute, Northwestern University, University of Texas, University of Virginia, University of Pennsylvania, Oregon Health & Science University, University of Chicago, and University of Utah. Among these, University of North Carolina and Dana Farber Cancer Institute most closely cooperated with Mayo Clinic. Fudan University cooperated with Soochow University, Shanghai Jiaotong University, Shanghai United Imaging Medical Company, Shanghai Key Laboratory of Radiation Oncology, and Fujian Medical University. Fudan University had a relatively close cooperation with Shanghai United Imaging Medical Company and Shanghai Key Laboratory of Radiation Oncology; however, it did not cooperate with foreign institutions. Sun Yat-Sen University cooperated with the University Texas MD Anderson Cancer Center, Zhengzhou University, Southern Medical University, and Fujian Medical University, among which the cooperation with Zhengzhou University and Southern Medical University was relatively close. Notably, both Fudan University and Sun Yat-Sen University cooperated with Fujian Medical University, but had low or no cooperation with foreign institutions.

Burst analysis showed that prominent foreign cancer institutions such as the University of Texas, National Cancer Center, and Mayo Clinic led past research on radiotherapy for rectal cancer. In recent years, the influence of institutions such as Fudan University, Sun Yat Sen University, Netherlands Cancer Institute, Maastricht University, and Chinese Academy of Medicine and Peking Union Medical College has increased. Three of these five institutions that recently emerged are in China, indicating the rapid development of research on rectal cancer radiotherapy in China (Figure 3B).

#### Analysis of authors and co-cited authors

**Figure 4:**
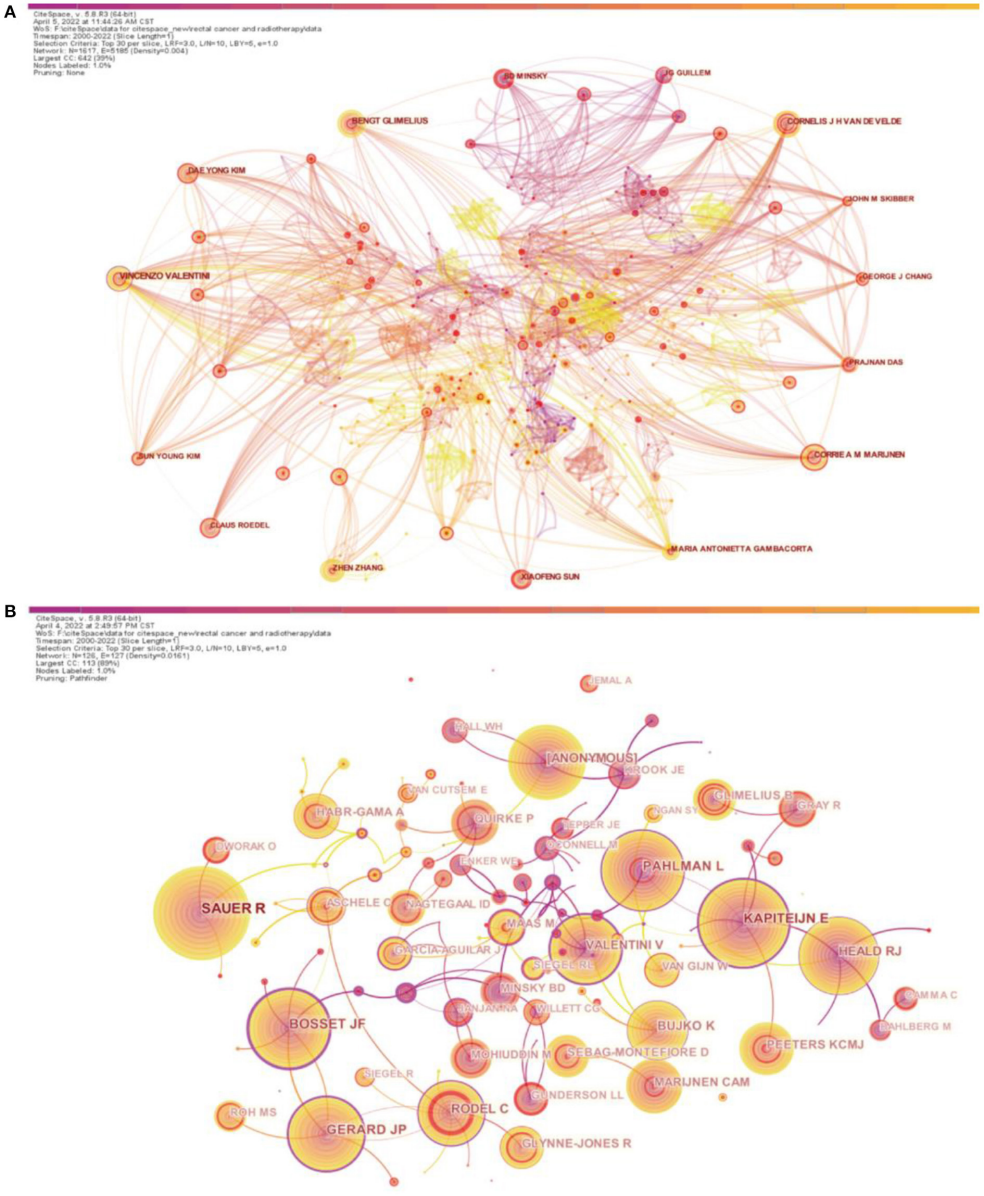
Analysis of authors and co-cited authors involved in research on radiotherapy for rectal cancer. **(A)** Network diagram showing author links (Timespan: 2000–2022; Slice Length: 1; Selection Criteria: Top30 per slice; Network: *N* = 1617, E = 5,185; Density: 0.004; Largest CC:642; Nodes Labeled:1.0%; Pruning: None). **(B)** Network diagram showing co-cited author links (Timespan: 2000–2022; Slice Length: 1; Selection Criteria: Top30 per slice; Network: N = 126, E = 127; Density: 0.0161; Largest CC:113; Nodes Labeled:1.0%; Pruning: Pathfinder).

More than 1,500 researchers have participated in studies related to radiation therapy for rectal cancer. The top three authors with the most published papers were Vincenzo Valentini (*n* = 41), Bengt Glimelius (*n* = 34), and Cornelis J.H. Van De Velde (*n* = 33) (Table 2). Among the top 10 co-cited authors (Table 1), R. Sauer (*n* = 1398) ranked first, followed by E. Kapiteijn (*n* = 971) and J.F. Bosset (*n* = 849) (Table 2). Figure 4 shows the relationship between authors or between co-cited authors. One node represents an author, and the size of the node represents the number of published articles: the higher the number of published articles, the larger the size of the node. With respect to the degree of cooperation between the authors, a wider line indicates a closer cooperation between two authors. Some authors had good cooperation with other authors, such as the Italian author Vincenzo Valentini, as shown by the large number of connections in the network diagram. This indicates that enhanced cooperation may help obtain more results. From the perspective of co-cited author centrality, J.F. Bosset from France had the highest centrality value (centrality, 0.73). This author published the results of several clinical trials on rectal cancer radiotherapy, most recently the results of a phase 3 clinical trial on FOLFIRINOX (oxaliplatin, irinotecan, fluorouracil and leucovorin) neoadjuvant chemotherapy and preoperative radiotherapy in patients with locally advanced rectal cancer in The Lancet in 2021 (14). He also collaborated more with experts in the field of rectal cancer radiotherapy, which might have helped achieve impactful results (Figure 4B).

**Table 2:** Ranking of authors and co-cited authors according to the number of published articles.

**Rank**	**Author**	**Count**	**Co-cited Author**	**Count**	**Centrality**
1	Vincenzo Valentini	41	Sauer R	1,398	0.06
2	Bengt Glimelius	34	Kapiteijn E	971	0.47
3	Cornelis J H Van De Velde	33	Bosset JF	849	0.73
4	Corrie A M Marijnen	32	Gerard JP	789	0.40
5	Dae Yong Kim	30	Pahlman L	756	0.39
6	Xiaofeng Sun	28	Rodel C	651	0.35
7	BD Minsky	26	Heald RJ	635	0.14
8	Zhen Zhang	26	Anonymous	575	0.08
9	Maria Antonietta Gambacorta	25	Valentini V	508	0.48
10	George J Chang	22	Bujko K	489	0.14

### Exploring the hotspots and evolution of research on radiotherapy for rectal cancer based on literature co-citations

#### Analysis of total number of citations

When two articles are cited together in other articles, they are considered to be in a co-citation relationship, and the frequency of co-citations is a measure of the degree of the relationship between the two articles (15). For statistical convenience, we selected the top 30 cited documents in a time slice of 1 year and constructed a co-citation network map. The citation number ranking of the articles was obtained by counting the number of citations (Table 3). The most cited article was by Sauer et al., published in the New England Journal of Medicine in 2004. This article compared preoperative chemoradiotherapy with postoperative chemoradiotherapy and concluded that preoperative chemoradiotherapy can improve local tumor control and is associated with reduced toxicity (16). The second most cited article was that by Kapiteijn et al., published in the New England Journal of Medicine in 2001. This article showed that short-term preoperative radiotherapy reduces the risk of local recurrence in patients with rectal cancer after total mesorectal resection (17). The third most cited article was the work by Bosset et al. published in the New England Journal of Medicine, which found that preoperative radiotherapy combined with fluorouracil chemotherapy had a significant benefit in the local control of rectal cancer (18). Notably, these top three cited articles were published in the New England Journal of Medicine, indicating the important influence of this journal in the field of rectal cancer radiotherapy. Two of the top five cited articles were studies by R. Sauer in Germany, indicating that this author made outstanding contributions in the field. In addition, the article with the highest centrality (centrality, 0.50) was that by Gerard et al. (20). They reported that preoperative chemoradiotherapy, despite a modest increase in acute toxicity and no effect on overall survival, significantly improved local control and was recommended for mid- and distal rectal T3-4, N0-2, M0 adenocarcinomas. A node with a centrality value of > 0.1 is considered a critical node, and this article had a centrality value of 0.5, indicating that it had a significant impact on subsequent studies on radiotherapy for rectal cancer (Table 3).

**Table 3:** Ranking of cited times of literature.

**Rank**	**Counts**	**Centrality**	**Year**	**Cited references**
1	287	0.07	2004	(16)
2	211	0.02	2001	(17)
3	205	0.01	2006	(18)
4	160	0.04	2012	(19)
5	158	0.5	2006	(20)

#### Analysis of co-citation burst characteristics

**Figure 5:**
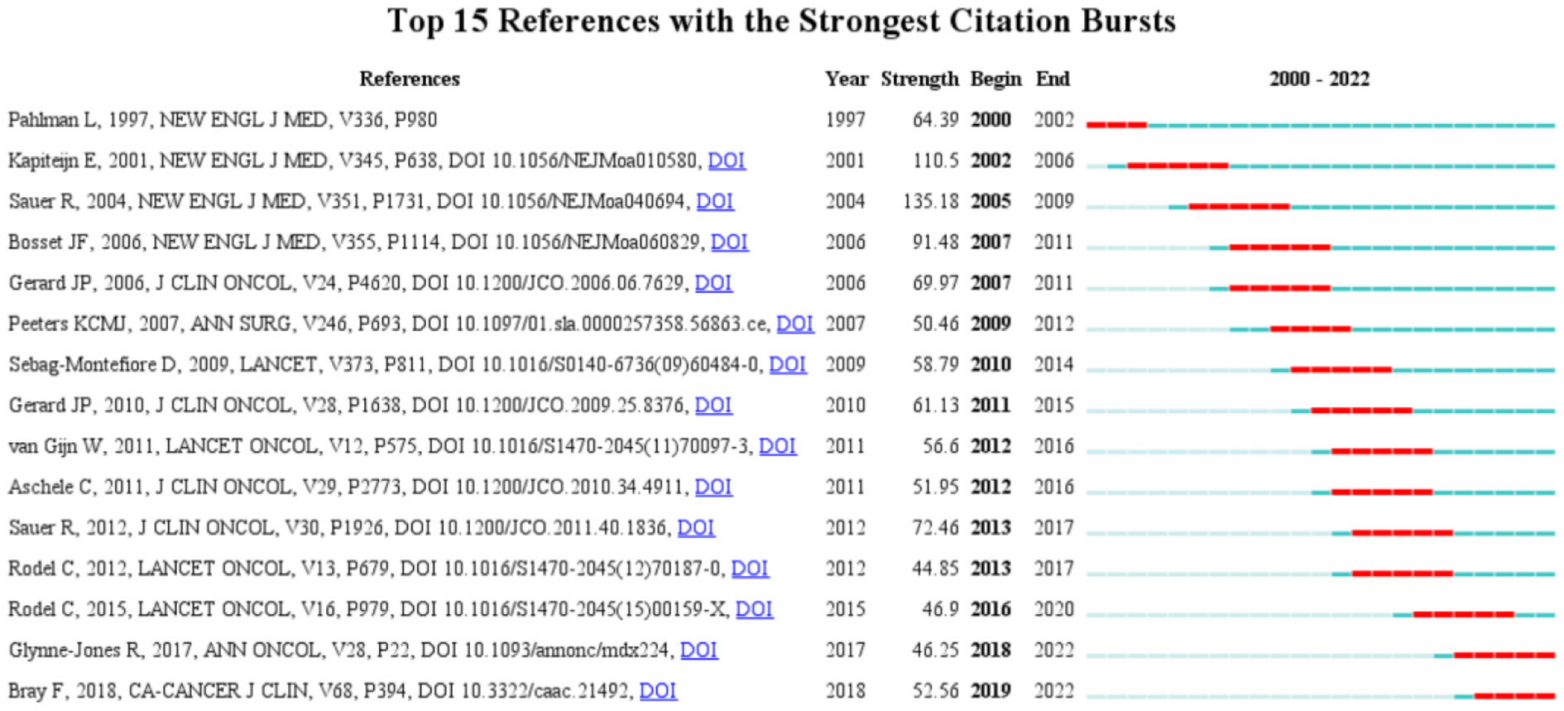
Top 15 articles related to radiation therapy for rectal cancer with burst period after 2000.

Burst analysis can identify articles that have attracted the attention of peer researchers and can help in the timely discovery of articles with a strong influence on future research (21). The collected articles were screened, and the top 15 studies according to the strength of citation bursts are shown in Figure 5. The top-ranked article in terms of citation burst strength was published in 2004, titled “Preoperative vs. postoperative chemoradiotherapy for rectal cancer”. The authors of the article concluded that preoperative chemoradiotherapy, compared with postoperative chemoradiotherapy, improved local control and was associated with reduced toxicity but not with improved overall survival (16). The article with the second strongest citation burst was published in 2001, titled “Preoperative radiotherapy combined with total mesorectal excision for resectable rectal cancer”, which concluded that short-term preoperative radiotherapy reduces the risk of local recurrence in patients with rectal cancer undergoing standardized total mesorectal excision (17). This was followed by an article titled “Chemotherapy with preoperative radiotherapy in rectal cancer” published in 2006, which concluded that the addition of preoperative or postoperative fluorouracil-based chemotherapy had no significant effect on the survival of patients with rectal cancer treated with preoperative radiotherapy. Chemotherapy, whether administered before or after surgery, offers significant benefits for local disease control (18). The top three articles with the strongest citation burst focused on the treatment plan before or after rectal cancer surgery. The conclusions of these studies suggest that preoperative chemoradiotherapy has significant benefits for local control.

According to the year of the citation burst, an article titled “Improved survival with preoperative radiotherapy in resectable rectal cancer” began to be associated with a surge of citations in 2000. This study concluded that a short-term regimen of high-dose preoperative radiotherapy may reduce local recurrence rates and improve survival in patients with resectable rectal cancer (22). The topic of “preoperative radiotherapy” became the focus of research in 2000–2002. The articles with citation bursts in the next few years were mainly focused on preoperative radiotherapy, and supported the conclusion that preoperative chemoradiotherapy improves local control but not overall survival (16–18, 20, 23, 24). An article that had citation bursts in 2010 reported the results of a multicentre randomized trial comparing preoperative radiotherapy and selective postoperative chemoradiotherapy for rectal cancer. The authors concluded that short-course preoperative radiotherapy is an effective treatment for patients with operable rectal cancer (23). Although the addition of oxaliplatin to fluorouracil-based adjuvant chemotherapy for colon cancer has been shown to improve disease-free survival and overall survival, no clear evidence existed at that time on the effectiveness of adding oxaliplatin to multimodal therapy for patients with locally progressive rectal cancer (25). In an article that had citation bursts in 2011, Gerard et al. evaluated the response of the primary tumor to preoperative radiotherapy with or without oxaliplatin. They concluded that oxaliplatin has unproven benefits in patients with advanced rectal cancer and that this drug should not be used in combination with radiation therapy (26). In an article that had citation bursts in 2012, R. Sauer et al. suggested that the addition of oxaliplatin to fluorouracil-based preoperative chemoradiotherapy significantly increased toxicity without affecting the primary tumor response, requiring a longer follow-up to assess the effect on efficacy endpoints (19). Although preoperative radiotherapy has been widely accepted, the use of oxaliplatin in combination with preoperative radiotherapy has been questioned. This question was answered by an article that had citation bursts in 2013, in which Rodel et al. reported the preliminary results of their phase 3 trial. The authors concluded that oxaliplatin can be incorporated into a modified fluorouracil-based combination therapy and that this regimen allows more patients to achieve pathological complete remission, compared with standard treatment (27). Rodel et al. published the final results of their phase 3 trial in 2015 (with citation bursts in 2016) and reported that oxaliplatin added to fluorouracil-based neoadjuvant radiotherapy and adjuvant chemotherapy significantly improved disease-free survival in patients with cT3-4 or cN1-2 rectal cancer. The articles with citation bursts in 2018 and 2019 focused on the clinical statistics of rectal cancer and the clinical guidelines for diagnosis and treatment. The burst time period has continued to the present. These two articles have strong guiding significance for the diagnosis and treatment of rectal cancer (28, 29).

Notably, among the top 15 articles with citation bursts, 5 articles were related to short-course radiotherapy (17, 22–24, 30). Short-course radiotherapy differs from conventional radiotherapy and usually involves 5 Gy × 5 irradiation (5). The results of studies performed in Sweden (31) and the Netherlands (30) showed that surgery after short-course radiotherapy can reduce the local recurrence rate compared with simple surgery and has advantages of lower treatment cost and better treatment convenience. For resectable rectal cancer, short-course radiotherapy has been shown to have similar local control and overall survival rates to conventional chemoradiotherapy, with lower toxicity (32). Thus, short-course radiotherapy is recommended as an alternative to neoadjuvant therapy. However, in the phase 3 studies, short-course radiotherapy resulted in lower tumor downstaging rates than conventional chemoradiotherapy (30, 31).

#### Analysis of the characteristics of co-citation clustering

**Figure 6:**
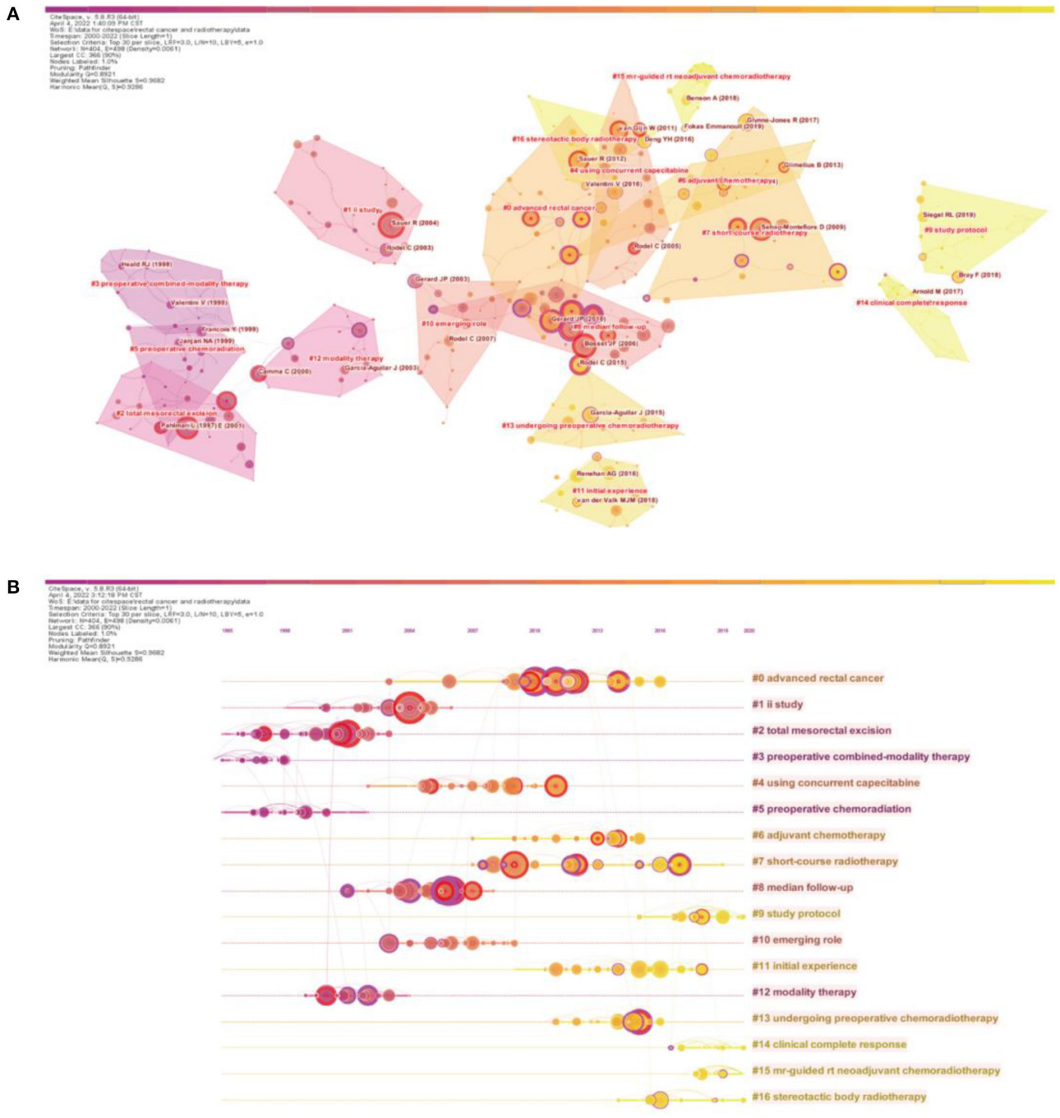
Analysis of the cited articles. **(A)** Knowledge map of the cited literature (Timespan: 2000–2022; Slice Length: 1; Selection Criteria: Top30 per slice; Network: *N* = 404, E = 498; Density: 0.0061; Largest CC:366; Nodes Labeled:1.0%; Pruning: Pathfinder; Modularity Q:0.8921; Weighted Mean Silhouette S:0.9682). **(B)** Time axis map of the cited literature (Timespan: 2000–2022; Slice Length: 1; Selection Criteria: Top30 per slice; Network: *N* = 404, E = 498; Density: 0.0061; Largest CC:366; Nodes Labeled:1.0%; Pruning: Pathfinder; Modularity Q:0.8921; Weighted Mean Silhouette S:0.9682).

In the cluster analysis, we found that the clustering themes of articles with citation bursts have considerably changed during the last 22 years. In Figure 6A, the lighter gray color represents the time period closer to the current time. The average year indicates the mean year of publication of the cited literature. The silhouette coefficient “S” reflects the amount of attention paid by researchers to the topic. S > 0.7 means that the clustering was convincing. The silhouette coefficients of the clusters in the figure were > 0.9 (Supplementary Table S2). The early themes were mainly about preoperative radiotherapy, and the studies mainly focused on patients with resectable rectal cancer and some patients with advanced rectal cancer (Figure 6 and Supplementary Table S2). Later, radiotherapy combined with chemotherapy regimens, including oxaliplatin, capecitabine, and bevacizumab, began to be studied in patients with unresectable rectal cancer. Subsequently, some new protocols, such as short-course radiotherapy, delayed surgery, x-ray brachytherapy, and stereotactic whole-body radiotherapy, began to be mentioned, and the study participants began to include patients with metastatic rectal cancer. This may be because researchers began to realize that the patient survival time mainly depends on distant metastases (24). Recently, magnetic resonance (MR)-guided neoadjuvant radiotherapy has begun to be investigated in patients with recurrent rectal cancer as well as in younger patients with colorectal cancer, which may be related to the increasing number of patients diagnosed with colorectal cancer at a young age (33).

The constructed co-citation network graph is presented based on the time nodes to obtain a timeline map (Figure 6B). The size of the circles in the map represents the number of citations: the larger the circle, the more citations the articles has received. The time map shows that research on rectal cancer radiotherapy was popular in the two periods of 2001–2007 and 2010–2013. In these two periods, some influential articles appeared, such as the article titled “Preoperative versus postoperative chemoradiotherapy for rectal cancer” by Sauer et al. in 2004, which is the most cited article in this field thus far (16). Eight of the top 10 cited articles in the field were from these two periods.

The largest cluster in the title clusters was “advanced rectal cancer”, reflecting its importance in research on radiotherapy for rectal cancer. The clusters with the largest silhouette coefficients were “ii study” and “study protocol”, indicating that these two clusters had the most explicit themes. The studies in these two clusters were mainly related to radiotherapy combined with chemotherapy and recurrent rectal cancer, reflecting the focus of research on this topic (Supplementary Table S2). In addition, as shown in Figure 6B, prior studies on rectal cancer radiotherapy were relatively concentrated in four clusters: #0 (advanced rectal cancer), #1 (ii study), #2 (total mesorectal excision), and #8 (median follow-up). Notably, clusters #15 (MR-guided neoadjuvant chemoradiotherapy) and #16 (stereotactic body radiotherapy [SBRT]), which have emerged recently, represent the latest technologies and protocols for rectal cancer radiotherapy.

### Exploring the hotspots and evolution of research on rectal cancer radiotherapy based on keywords

#### Analysis of keyword co-occurrence clustering

**Figure 7:**
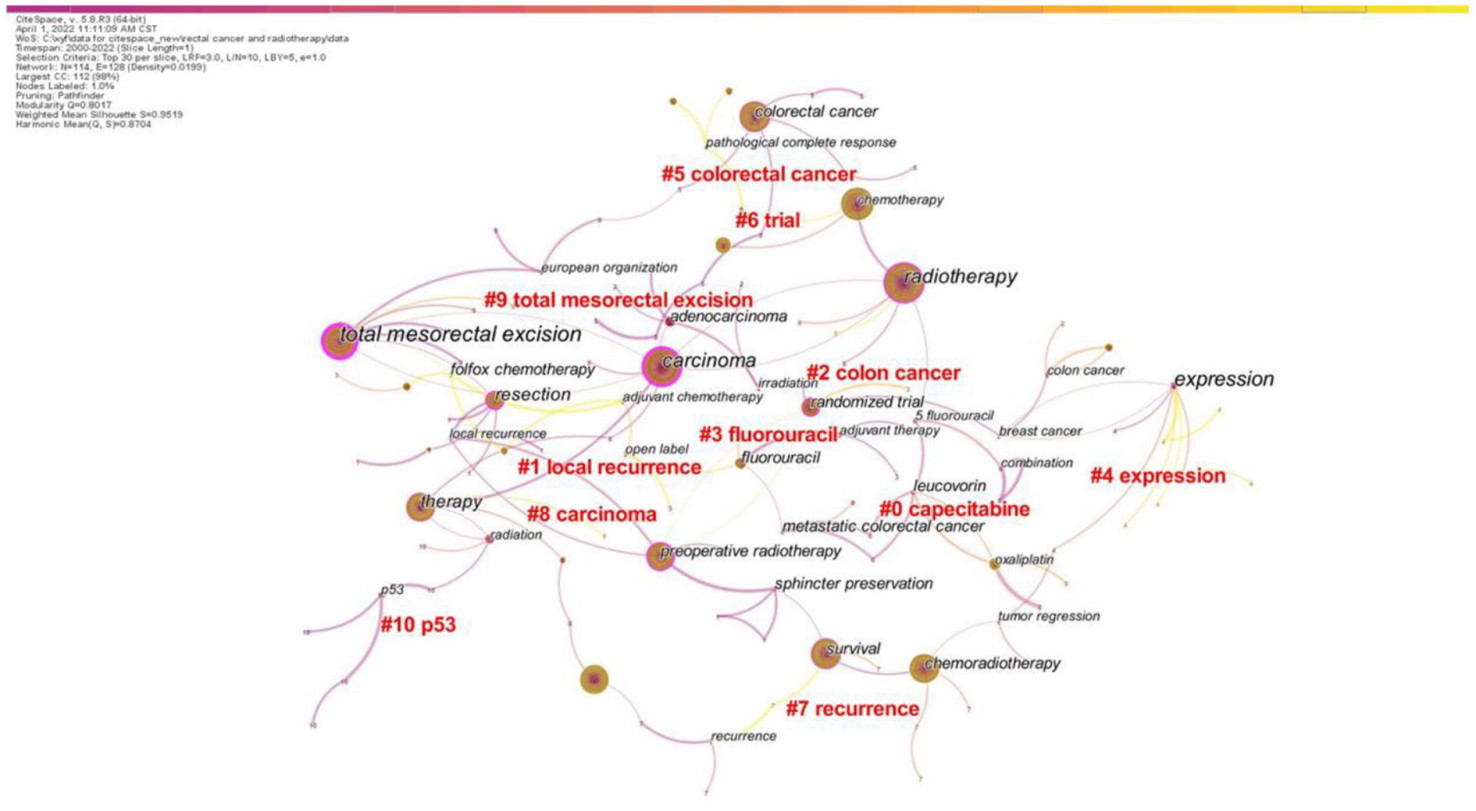
CiteSpace visualization map of the keyword clustering analysis (Timespan: 2000–2022; Slice Length: 1; Selection Criteria: Top30 per slice; Network: *N* = 114, E = 128; Density: 0.0199; Largest CC:112; Nodes Labeled:1.0%; Pruning: Pathfinder; Modularity Q:0.8017; Weighted Mean Silhouette S:0.9519).

Keyword co-occurrence analysis can reflect the hotspots of research in a field. This analysis was performed on the 5,372 articles published between January 2000 and January 2022. The time slice was set to 1 year, and the top 30 keywords that appeared most frequently in each slice were selected. After merging similar keywords (“radiation therapy” was merged with “radiotherapy”, “chemoradiation” was merged with “chemoradiotherapy”) and using a Pathfinder network for tailoring (PFNET pathfinding network), a keyword co-occurrence map was generated (Figure 7). The nodes represent keywords, and the size of each node corresponds to the co-occurrence frequency of the keywords. The colors of the nodes and connecting lines represent the chronological order: color from dark to light indicates time from far to near. The top 10 keywords with the highest frequency were “radiotherapy”, “carcinoma”, “chemoradiotherapy”, “total mesorectal excision”, “survival”, “colorectal cancer”, “chemotherapy”, “surgery”, “preoperative radiotherapy”, and “therapy”. Among them, “radiotherapy” corresponded to the largest node, indicating that radiotherapy plays an important role in the field of rectal cancer treatment. Preoperative or postoperative radiation therapy has been shown to reduce the risk of local recurrence in patients with operable rectal cancer (23, 34). The keywords “tumor” and “total mesorectal resection” had a large centrality, indicating that these keywords were the key nodes in the graph. Since its introduction in the 1980s, total mesorectal excision has revolutionized the surgical treatment of rectal cancer. This surgical method not only considerably improves patient outcomes but also reduces local recurrence rates, preserves sphincter function, and decreases the incidence of complications (35). Currently, preoperative radiotherapy followed by total mesorectal resection is the standard treatment for patients with locally advanced rectal cancer (36).

Table 4 shows the main clusters of keywords. The first eight clusters were all ≥ 10 in size and possessed high silhouette coefficients, suggesting successful clustering with high confidence. Among the four largest clustering categories, “local recurrence” had the largest profile coefficient and the earliest mean study year. This indicates that the impact of local recurrence on patients with rectal cancer has long been a research concern. With the constant development of surgical strategies and adjuvant/neoadjuvant treatments, the local recurrence rate of rectal cancer has considerably decreased. However, postoperative recurrence and distant metastasis remain the main causes of treatment failure (37, 38). In addition, the average burst time of “capecitabine” was in the early period, which indicates that the combination of radiotherapy and chemotherapy for rectal cancer has long been studied. The attention to “trial” and “expression” has increased over time, whereas the average burst time of “expression” was in the most recent period. These findings imply that clinical trials and mechanistic studies may be hot research topics in the field of rectal cancer treatment in recent years.

**Table 4:** Main clusters of keywords.

**Cluster ID**	**Size**	**Silhouette**	**Mean year**	**Label (LLR)**
0	12	0.915	2003	Capecitabine
1	12	0.965	2000	Local recurrence
2	12	0.946	2002	Colon cancer
3	12	0.96	2008	Fluorouracil
4	11	0.95	2012	Expression
5	10	1.00	2001	Colorectal cancer
6	10	1.00	2008	Trial
7	10	0.844	2005	Recurrence

#### Analysis of keyword bursts

**Figure 8:**
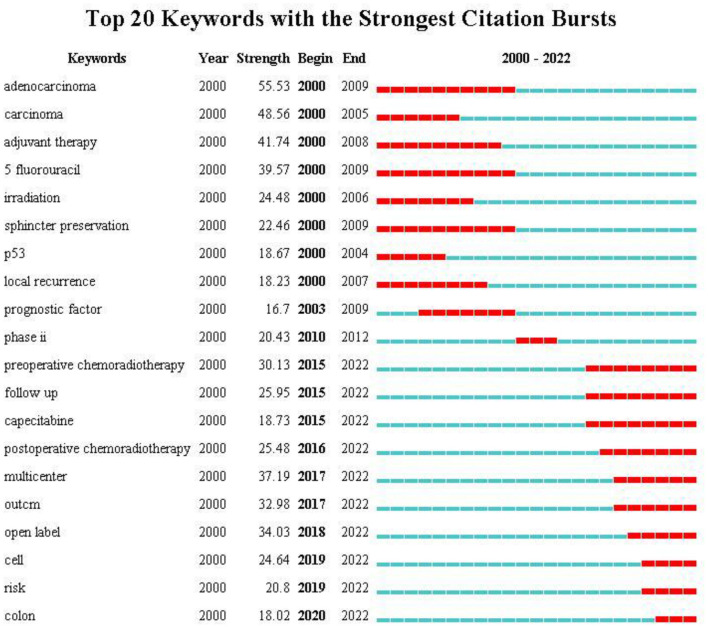
Top 15 keywords related to radiation therapy for rectal cancer with burst period after 2000.

Keyword burst analysis not only can reflect the changes in research hotspots in a certain field but can also predict future research trends. As shown in Figure 8, the change in keyword bursts over the past 22 years demonstrated the evolution of hotspots in rectal cancer research. Among the top 20 keywords with bursts, “adenocarcinoma” had the highest burst strength (strength, 55.53) and longest burst duration (10 years). This indicates that adenocarcinoma has an important role in the study of rectal cancer, which may be related to the fact that it is the most common pathological type of rectal cancer. The burst time of “fluorouracil” and “sphincter preservation” also lasted from 2000 to 2009, suggesting that researchers have focused on adjuvant chemotherapy for rectal cancer and postoperative anal sphincter preservation. Moreover, early research hotspots also included the expression of the oncogene p53 and local recurrence as prognostic indicators. The next two keywords that emerged were “prognostic factors” and “phase II”, which may indicate the emergence of clinical trials.

The remaining 10 keywords that have received more attention from after 2015 to the present time are the focus of current studies in the field of rectal cancer radiotherapy. Among them, “multicenter” and “open label” had the highest burst strength, indicating that the research activity was more concentrated on clinical trials. Clinical trials have guiding significance in the formulation of clinical treatment strategies. Another research hotspot emerged related to preoperative and postoperative radiotherapy, with bursts beginning in 2015 and 2016, respectively, and continuing to the present time. On the basis of numerous clinical trials, total mesorectal resection after preoperative 5-fluorouracil-based neoadjuvant radiotherapy has become the standard treatment for locally progressive rectal cancer, significantly reducing the rate of local recurrence but not the risk of metastatic recurrence (36, 39). Postoperative radiotherapy is less effective than preoperative radiotherapy in terms of sphincter preservation, toxicity, and overall treatment compliance (40). Therefore, the optimal indications for postoperative adjuvant therapy remain to be determined. Moreover, as a radiosensitiser and a safe and effective alternative to 5-fluorouracil, the role of capecitabine in adjuvant or neoadjuvant radiotherapy regimens may be a new hot topic (41, 42).

## Discussion

In this study, a systematic literature search of the WoSCC database was conducted for articles on radiotherapy for rectal cancer published between 2000 and 2022. After excluding studies that did not meet the screening criteria, the scientometric analysis included 5,372 English-language papers from 130 countries. The research results and progress in this field were quantitatively and visually assessed using the CiteSpace econometric analysis software.

Articles associated with research on rectal cancer chemotherapy over the last 22 years were analyzed from different dimensions and perspectives. With respect to the volume of publications, the number of articles in the field of rectal cancer has increased every year, indicating that increasing attention has been paid to research related to rectal cancer radiotherapy. In terms of countries of publication, European countries, Japan, Korea, Australia conducted the earlier studies on rectal cancer radiotherapy, and the United States has the highest number of publications in this field. The analysis of institutional bursts showed that foreign institutions have ushered the development of this field in the past. However, of the five institutions that emerged recently, three are in China. Although research in this field started late in China, rapid developments have been achieved. The analysis of authors and co-cited authors showed that the Italian author Vincenzo Valentini ranked first in terms of the number of published articles, reflecting his extensive research in the field, whereas the German author R. Sauer ranked first in terms of co-citations, indicating his significant influence in the field of radiotherapy for rectal cancer. In the co-citation analysis, R. Sauer's article published in the New England Journal of Medicine, which established the importance of neoadjuvant radiotherapy in the treatment of locally progressive rectal cancer, was the most frequently cited article (5).

Through a literature clustering analysis, we found that the clustering topics of burst literature have considerably changed in recent years, which is mainly reflected in three aspects of rectal cancer radiotherapy: objects, types, and methods. In the past, the study participants included patients with resectable rectal cancer and some patients with advanced rectal cancer. More recently, studies gradually included patients with unresectable rectal cancer, patients with metastatic rectal cancer, patients with recurrent rectal cancer, and young patients with rectal cancer. Radiotherapy types have changed from postoperative radiotherapy to preoperative radiotherapy and now to preoperative radiotherapy combined with chemotherapy. Radiotherapy methods for rectal cancer has changed from conventional radiotherapy to brachytherapy and to the current stereotactic radiotherapy and MR-guided neoadjuvant radiotherapy. Notably, among these, the radiotherapy approach was the fastest evolving method. SBRT uses stereotactic technology for radiation therapy to improve positioning and positional accuracy (43), whereas three-dimensional conformal radiotherapy and intensity-modulated radiotherapy both involve the application of stereotactic technology (44). In rectal cancer, both local recurrence and distant metastasis are important issues associated with substantial morbidity and mortality (45). SBRT has been successfully applied in the treatment of pelvic recurrence of rectal cancer and liver metastasis of rectal cancer (46, 47). Compared with conventional external beam therapy, this technique has a smaller dilated margin and greater conformity, and is a safer way to minimize repeat irradiation of normal structures (45). The introduction of MR-guided radiotherapy has led to a new era in radiation oncology, offering the possibility of providing daily online adaptive radiotherapy and acquiring images with higher soft-tissue contrast (48–50). MR-guided radiotherapy is a type of image-guided radiotherapy and is a four-dimensional radiotherapy technique that adds the concept of temporal sequencing to the three-dimensional radiotherapy technique (51). Image-guided radiotherapy is used to define target and non-target structures, design and validate treatment plans, and reduce positional and treatment errors with the aid of modern imaging methods such as computed tomography/MR imaging/positron emission tomography or ultrasound (51). Both SBRT and MR-guided radiotherapy are aimed at improving the precision of radiotherapy and reducing its toxic effects, and both are representative precision radiotherapy techniques.

Keyword co-occurrence and burst analyses have enabled the evaluation of hot research topics and emerging research areas in the field of rectal cancer radiotherapy over the past 22 years. Early researchers focused more on total mesorectal resection, combined radiotherapy and chemotherapy, local recurrence, sphincter preservation, and treatment with 5-fluorouracil. Later, researchers gradually explored a system of rectal cancer treatments based on surgery and supplemented by radiotherapy to reduce postoperative complications and improve prognosis. In recent years, the keywords of interest included “p53”, “expression”, “multicenter”, “open trial”, “preoperative radiotherapy”, and “postoperative radiotherapy”, which indicate that mechanistic studies, clinical trials, and neoadjuvant/adjuvant radiotherapy studies may be the recent hotspots in the field of rectal cancer. Currently, an increasing number of clinical trials are exploring the outstanding issues in the field of rectal cancer radiotherapy, such as the dose of radiotherapy, the optimal indications, and the timing of adjuvant therapeutic interventions, to guide the clinical development of a more rational individualized treatment strategy.

Clinical trials related to rectal cancer radiotherapy are of great significance for promoting the development of clinical treatment. Previous clinical trials discussed the toxicity and survival outcomes of neoadjuvant radiotherapy, the choice of short- and long-course radiotherapy. Whether preoperative radiotherapy can increase patient survival remains controversial. Early studies have shown survival benefit of preoperative radiotherapy (31, 52), but recent studies do not support this conclusion (19, 23, 53, 54). Notably, it has become a consensus that preoperative radiotherapy can reduce the local recurrence rate and improve the surgical resection rate, tumor local control rate and sphincter preservation rate of rectal cancer (14, 55, 56). There are two common methods of neoadjuvant radiotherapy: conventionally fractionated long-course radiotherapy (45–50.4 Gy/25-28 days) followed by delayed surgery and hypofractionated short-course radiotherapy (25 Gy/5 days) followed by immediate surgery (57). Studies have shown that both radiation regimens have pros and cons (57, 58). Short-course radiotherapy is simple, convenient and easy to implement. Its advantages are that the single dose of radiation is large, the radiotherapy cycle is short, and surgery is performed after 1 week of radiotherapy, which significantly shortens the preoperative treatment time, and decreases patients' cost. The disadvantage is that there is not enough time for tumor regression after short-course radiotherapy, and the effect of tumor downstaging is not obvious. It is not suitable for patients who are evaluated for unresectable before treatment. In order to improve its defects, recent research mainly focus on prolonging the interval between radiotherapy and surgery, or delaying surgery after short-course radiotherapy and sequential chemotherapy (59, 60). In addition, other clinical trials are still working on unresolved issues in the field of rectal cancer radiotherapy, such as the dose of radiotherapy, the intervention of immune drugs or new chemotherapy drugs, the best indications, and the timing of adjuvant therapy intervention. Undoubtedly, these clinical trials are important for guiding the clinical formulation of more reasonable individualized treatment strategies.

To our knowledge, this study is the first bibliometric analysis focusing on research in the field of rectal cancer radiotherapy. The data downloaded from the WoSCC database covered the vast majority of articles in the field of rectal cancer radiotherapy. Moreover, our data analysis was relatively objective and comprehensive, clearly demonstrating the current state of research in this field. However, this study had some limitations. First, this study included original articles published between 2000 and 2022 and indexed in the WoSCC database. The literature included in our study may not be exhaustive, as books, conference abstracts, and other types of publications were excluded from the document screening process. Second, only articles written in English were included in our analysis, which might have introduced some language bias. Third, recently published high-quality articles may not have received due attention because of low citation rates, which indicates the importance of future research updates. Therefore, the articles included in our analysis may not adequately reflect all studies on radiotherapy treatment for rectal cancer; however, our analysis covered the vast majority of articles in the field of rectal cancer radiotherapy, which is sufficient for the analysis of research trends and hotspots.

## Conclusion

Starting from the radiotherapy of rectal cancer, we use bibliometrics to visualize the research progress and emerging research hotspots in the field of rectal cancer radiotherapy, and provide researchers with a comprehensive landscape. In the past 20 years, a large number of high-quality studies on rectal cancer radiotherapy have emerged, which has promoted the development of clinical rectal cancer treatment. Through bibliometric analysis, we believe that rectal cancer radiotherapy will remain a hot research topic in the future and cooperations between countries and institutions will be closer. Research will continue to be mainly in the form of clinical trials, and basic research on mechanisms related to radiotherapy for rectal cancer will also expand. The research participants will be more diversified in the future, including patients with metastatic rectal cancer, patients with recurrent rectal cancer, and young patients with rectal cancer. The research themes will also keep pace with the times, whereas new treatment modalities, such as radiotherapy combined with different chemotherapeutic agents, SBRT, and MR-guided neoadjuvant radiotherapy, will continue to be explored. In conclusion, we hope this article can provide some inspiration for relevant researchers and clinicians.

## Data availability statement

The original contributions presented in the study are included in the article/Supplementary material, further inquiries can be directed to the corresponding author.

## Author contributions

CQ contributed to the conception and design of this paper. YX, MQ, and WH downloaded and organized related papers. YX, MQ, WH, and SH contributed to all tables and figures and the main manuscript. CT, FN, XJ, DW, ML, and QL revised and supplemented the manuscript. All authors read and approved the final manuscript.

## Funding

This study was supported by the National Natural Science Foundation of China (NSFC-U1504818), Science and Technology Foundation of Henan Province (172102310152, SBGJ202002097, and 192102310099), Natural Science Foundation of Henan Province (182300410359), Henan Provincial Education Fund (19A320020), Postgraduate Cultivating Innovation and Quality Improvement Action Plan of Henan University (SYL20060192, SYLYC2022141 and SYLJD2022009).

## Conflict of interest

The authors declare that the research was conducted in the absence of any commercial or financial relationships that could be construed as a potential conflict of interest.

## Publisher's note

All claims expressed in this article are solely those of the authors and do not necessarily represent those of their affiliated organizations, or those of the publisher, the editors and the reviewers. Any product that may be evaluated in this article, or claim that may be made by its manufacturer, is not guaranteed or endorsed by the publisher.
