# Supplementary material for: Global status of research on radiotherapy for rectal cancer: A bibliometric and visual analysis
Source: Front Public Health. 2022 Aug 8;10:962256. doi: 10.3389/fpubh.2022.962256 (PMC9393343; doi:10.3389/fpubh.2022.962256)
Supplement: Supplementary file 1 [file Data_Sheet_1.docx]

**Strategies for literature search in radiotherapy for rectal cancer：**

Databases: SCI-EXPANDED, CPCI-S, CPCI-SSH, BKCI-S, BKCI-SSH

#1: TI= ("rectal neoplasm") OR TI= ("rectal tumor") OR TI= ("rectal cancer") OR TI= ("rectum cancer") OR TI= ("colorectal cancer") OR TI= ("colorectal carcinoma") OR TI= ("colorectal tumor") OR AB= ("rectal neoplasm") OR AB= ("rectal tumor") OR AB= ("rectal cancer") OR AB= ("rectum cancer") OR AB= ("colorectal cancer") OR AB= ("colorectal carcinoma") OR AB= ("colorectal tumor")

#2: TI=(radiotherapy) OR TI=(radiation) OR AB=(radiotherapy) OR AB=(radiation)

((#1 AND #2) AND DT=(Article)) AND LA=(English)

DOP: 2000-01-01 to 2022-01-01

Table S1 Annual growth rate of rectal cancer radiotherapy publications.

| Year | Number (publication) | Annual growth rate (%) |
| --- | --- | --- |
| 2000 | 90 | - |
| 2001 | 108 | 20.00 |
| 2002 | 126 | 16.67 |
| 2003 | 124 | -1.59 |
| 2004 | 134 | 8.06 |
| 2005 | 184 | 37.31 |
| 2006 | 191 | 3.80 |
| 2007 | 209 | 9.42 |
| 2008 | 194 | -7.18 |
| 2009 | 222 | 14.43 |
| 2010 | 231 | 4.05 |
| 2011 | 253 | 9.52 |
| 2012 | 255 | 0.79 |
| 2013 | 259 | 1.57 |
| 2014 | 309 | 19.31 |
| 2015 | 291 | -5.82 |
| 2016 | 320 | 9.97 |
| 2017 | 313 | -2.19 |
| 2018 | 348 | 11.18 |
| 2019 | 369 | 6.03 |
| 2020 | 378 | 2.43 |
| 2021 | 464 |  |

**Table S2 Overview of main clusters cited in the literature.**

| **Cluster id** | **Mean year** | **Silhouette** | **Top terms log-likelihood ratio, p-level（LLR）** |
| --- | --- | --- | --- |
| 0 | 2011 | 0.965 | advanced rectal cancer (952.66, 1.0E-4); Chinese rectal cancer patient (509.64, 1.0E-4); high risk (492.18, 1.0E-4); pilot study (435.04, 1.0E-4); phase ii trial (419.79, 1.0E-4) |
| 1 | 2002 | 1 | ii study (602.14, 1.0E-4); weekly oxaliplatin (565.21, 1.0E-4); oral capecitabine (438.98, 1.0E-4); patients irradiation (368.15, 1.0E-4); bevacizumab oxaliplatin (363.1, 1.0E-4) |
| 2 | 1999 | 0.961 | total mesorectal excision (678.77, 1.0E-4); Swedish rectal cancer trial (674.36, 1.0E-4); short-term preoperative radiotherapy (656.8, 1.0E-4); multicenter randomized trial (626.94, 1.0E-4); advanced rectal cancer (625.94, 1.0E-4) |
| 3 | 1996 | 0.951 | preoperative combined-modality therapy (108.3, 1.0E-4); pretreatment clinical finding (97.46, 1.0E-4); receiving preoperative radiation (97.46, 1.0E-4); specialty center (86.61, 1.0E-4); advanced recurrent rectal cancer (75.77, 1.0E-4) |
| 4 | 2007 | 0.969 | using concurrent capecitabine (331.19, 1.0E-4); pathologic nodal status (326.53, 1.0E-4); pilot feasibility study (317.19, 1.0E-4); t3 mid (307.85, 1.0E-4); to-stage comparison (307.85, 1.0E-4) |
| 5 | 1998 | 0.943 | preoperative chemoradiation (321.73, 1.0E-4); conformal preoperative endorectal brachytherapy treatment (222.57, 1.0E-4); continuous infusion (210.45, 1.0E-4); t3-4nx rectal cancer (208.94, 1.0E-4); radiotherapy concomitant (208.94, 1.0E-4) |
| 6 | 2012 | 0.918 | adjuvant chemotherapy (431.07, 1.0E-4); postoperative chemotherapy (336.62, 1.0E-4); consensus statement (299.79, 1.0E-4); 16th annual western Canadian (299.79, 1.0E-4); Saskatoon Saskatchewan (299.79, 1.0E-4) |
| 7 | 2012 | 0.981 | short-course radiotherapy (2316.63, 1.0E-4); delayed surgery (1661.29, 1.0E-4); preoperative short-course (667.91, 1.0E-4); valproic acid (465.84, 1.0E-4); t3 rectal cancer (298.98, 1.0E-4) |
| 8 | 2004 | 0.95 | short-course radiotherapy (409.32, 1.0E-4); median follow-up (399.3, 1.0E-4); future direction (389, 1.0E-4); radiation therapy (303.45, 1.0E-4); staging prognostic factor (288.58, 1.0E-4) |
| 9 | 2018 | 1 | study protocol (685.76, 1.0E-4); colorectal cancer (511.61, 1.0E-4); multicentre open-label parallel-arm (455.19, 1.0E-4); controlled study (455.19, 1.0E-4); recurrent rectal cancer (403.25, 1.0E-4) |
| 10 | 2006 | 0.959 | emerging role (285.96, 1.0E-4); targeted agent (285.96, 1.0E-4); corgi-l study (262.69, 1.0E-4); multicentre phase ii trial (262.69, 1.0E-4); unresectable colorectal cancer (262.69, 1.0E-4) |
| 11 | 2014 | 0.982 | initial experience (794.37, 1.0E-4); x-ray brachytherapy (494.92, 1.0E-4); organ preservation (406.36, 1.0E-4); advanced rectal cancer (368.02, 1.0E-4); local regrowth (344.31, 1.0E-4) |
| 12 | 2001 | 0.992 | modality therapy (614.34, 1.0E-4); rectal carcinoma (400.07, 1.0E-4); posttreatment tnm staging (260.14, 1.0E-4); prognostic indicator (260.14, 1.0E-4); multidisciplinary rectal cancer treatment (259.58, 1.0E-4) |
| 13 | 2013 | 0.968 | undergoing preoperative chemoradiotherapy (321.45, 1.0E-4); new strategies (249.26, 1.0E-4); seom clinical guideline (245.41, 1.0E-4); routine practice (243.83, 1.0E-4); vailable evidence (243.83, 1.0E-4) |
| 14 | 2018 | 0.99 | clinical complete response (132.61, 1.0E-4); recurrent rectal adenocarcinoma (117.38, 1.0E-4); t2n0m0 distal rectal cancer (114.43, 1.0E-4); total mesorectal excision v (114.43, 1.0E-4); nonoperative management (108.55, 1.0E-4) |
| 15 | 2019 | 0.957 | mr-guided rt neoadjuvant chemoradiotherapy (214.77, 1.0E-4); predictive model (214.77, 1.0E-4); neoplastic diseases (206.43, 1.0E-4); theragnostic utilities (206.43, 1.0E-4); young-onset colorectal cancer (198.11, 1.0E-4) |
| 16 | 2017 | 0.991 | stereotactic body radiotherapy (658.09, 1.0E-4); positive lateral pelvic lymph node (494.05, 1.0E-4); oligometastatic colorectal cancer (483.76, 1.0E-4); lateral lymph node metastasis (317.85, 1.0E-4); distal rectal cancer patient (269.12, 1.0E-4) |
